# zflncRNApedia: A Comprehensive Online Resource for Zebrafish Long Non-Coding RNAs

**DOI:** 10.1371/journal.pone.0129997

**Published:** 2015-06-11

**Authors:** Heena Dhiman, Shruti Kapoor, Ambily Sivadas, Sridhar Sivasubbu, Vinod Scaria

**Affiliations:** 1 GN Ramachandran Knowledge Center for Genome Informatics, CSIR–Institute of Genomics and Integrative Biology, Mathura Road, Delhi, 110020, India; 2 Academy of Scientific and Innovative Research (AcSIR), Anusandhan Bhawan, Delhi, 110001, India; 3 Genomics and Molecular Medicine, CSIR Institute of Genomics and Integrative Biology, Mathura Road, Delhi, 110025, India; Medical College of Wisconsin, UNITED STATES

## Abstract

Recent transcriptome annotation using deep sequencing approaches have annotated a large number of long non-coding RNAs in zebrafish, a popular model organism for human diseases. These studies characterized lncRNAs in critical developmental stages as well as adult tissues. Each of the studies has uncovered a distinct set of lncRNAs, with minor overlaps. The availability of the raw RNA-Seq datasets in public domain encompassing critical developmental time-points and adult tissues provides us with a unique opportunity to understand the spatiotemporal expression patterns of lncRNAs. In the present report, we created a catalog of lncRNAs in zebrafish, derived largely from the three annotation sets, as well as manual curation of literature to compile a total of 2,267 lncRNA transcripts in zebrafish. The lncRNAs were further classified based on the genomic context and relationship with protein coding gene neighbors into 4 categories. Analysis revealed a total of 86 intronic, 309 promoter associated, 485 overlapping and 1,386 lincRNAs. We created a comprehensive resource which houses the annotation of lncRNAs as well as associated information including expression levels, promoter epigenetic marks, genomic variants and retroviral insertion mutants. The resource also hosts a genome browser where the datasets could be browsed in the genome context. To the best of our knowledge, this is the first comprehensive resource providing a unified catalog of lncRNAs in zebrafish. The resource is freely available at URL: http://genome.igib.res.in/zflncRNApedia

## Introduction

Long non-coding RNAs (lncRNAs) are a recently discovered class of non protein coding transcripts encoded by many metazoan genomes [1]. Members of this class have been largely annotated in the recent years following the transcriptome annotation of metazoans using deep sequencing approaches [2–5]. By definition, lncRNAs are transcripts with a length of more than 200 nucleotides and with no obvious potential to translate to a functional protein [6]. In contrast to their shorter and well studied counterparts like microRNAs, a majority of the lncRNAs have not been functionally characterized. Nevertheless a handful of lncRNAs which have been characterized and extensively studied in the recent years provide us with a view of their roles in regulating and modulating critical processes in the cell. lncRNAs are presently known to function in a variety of ways, including recruitment of chromatin remodelers, antisense regulation of messenger RNAs, serving as scaffolds for recruitment of regulatory proteins and sequestration of small regulatory RNAs, apart from serving as substrates for biogenesis of small non-coding RNAs [7–10]. In addition, recent evidence suggests their association and mechanistic role in various human diseases including cancer, and has been suggested to serve as potential therapeutic targets [11, 12].

Systematic efforts have been made to curate the lncRNAs encoded by many metazoan genomes including human and other model organisms. Although a popular model organism to study human diseases, there has been a paucity of a unified catalog of lncRNAs in zebrafish. A number of resources provide information on a subset of lncRNAs in zebrafish which include ZFIN [13], lncRNAdb [14] and lncRNAtor [15] and Z-SEQ [16]. These databases catalog unique and spatiotemporally distinct subsets of the lncRNAs in zebrafish. For example, ZFIN stores data for genetic, genomic and developmental information related to zebrafish, lncRNAdb and lncRNAtor report few well-validated class of lncRNAs, while Z-SEQ catalogs lincRNAs from a single study [16]. The paucity of a unified catalog has limited a holistic understanding of lncRNAs and comparison of their spatiotemporal expression patterns.

The recent transcriptome analysis of zebrafish using deep sequencing approaches has uncovered a hitherto unknown set of transcripts including a number of novel long non-coding RNAs. The major proportions of the lncRNAs known to date in zebrafish have come from three large studies, which have extensively used next-generation sequencing approaches to uncover the lncRNome of zebrafish [16–18]. A well curated and biologically oriented resource for lncRNAs is required for a systematic study of these transcripts. In the present manuscript, we report zflncRNApedia, a comprehensive and unified resource for lncRNAs in zebrafish. The resource provides an insight into the genomic context, expression and regulation of each of the lncRNAs identified in 5 different tissues and 10 developmental time points. To the best of our knowledge, this is the first and only resource providing a unified view of the zebrafish lncRNome and their spatiotemporal expression across developmental time-points and adult tissues. The resource is available at URL: http://genome.igib.res.in/zflncRNApedia

## Materials and Methods

**Fig 1:**
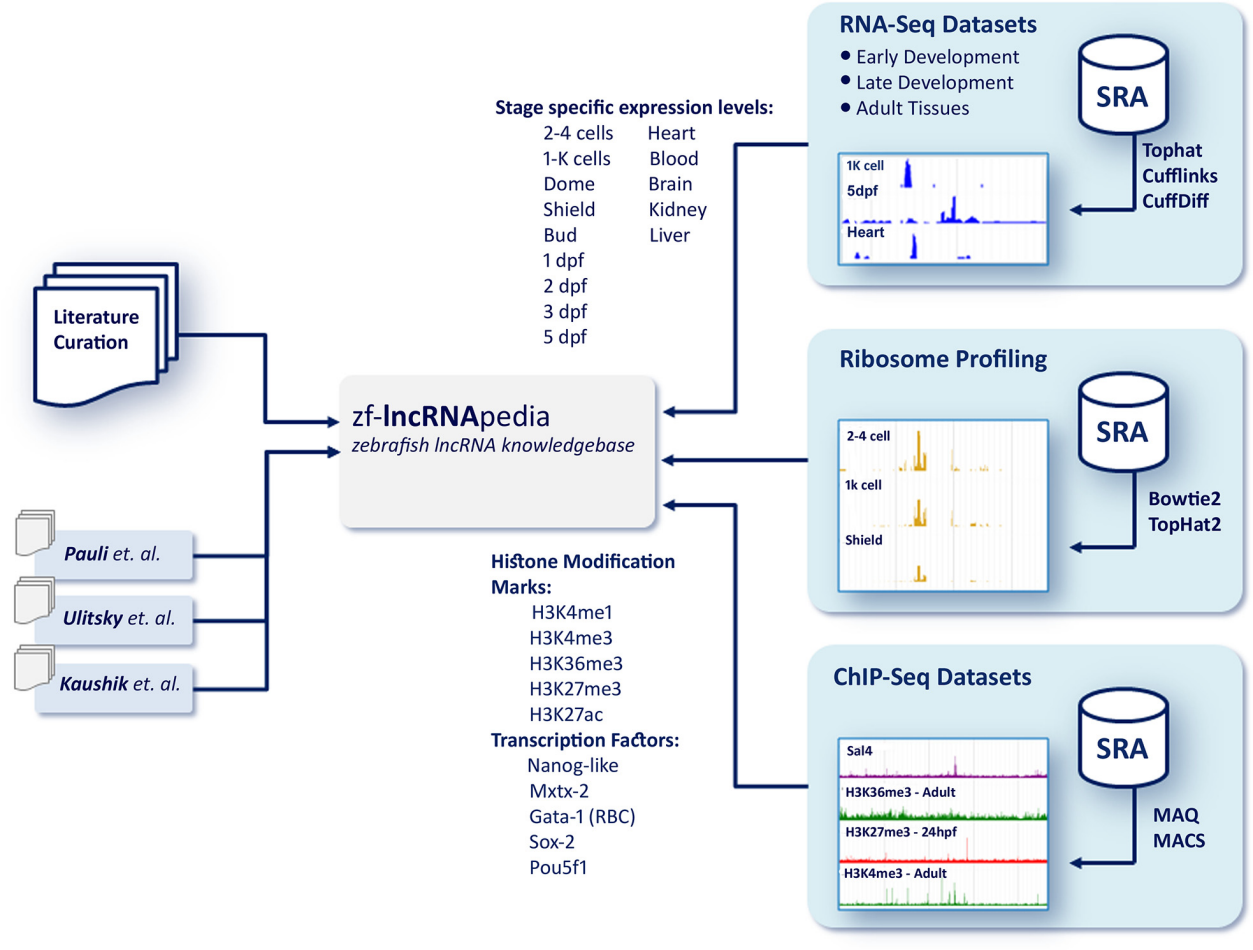
Workflow detailing data curation and methodologies involved in building the resource.

Towards providing a comprehensive resource of lncRNA annotation a number of independent datasets have been integrated. This include the histone modification marks towards understanding the promoter architecture and regulation, expression levels recomputed from the raw datasets, open reading frame predictions and ribosome profiling data sets towards understanding the coding potential of transcripts and genomic variations towards understanding the variability and mutant information to prioritize potential mutants for in-depth studies. The entire workflow for data curation is summarized in Fig 1. Descriptions of the datasets and methods are detailed below.

### Compendium of zebrafish lncRNAs

The lncRNA annotations were independently derived from manual curation of data from published literature and supplementary resources [16–18]. The lncRNA annotations and their genomic loci were collated. The bulk of annotations came from the three recent RNA-Seq datasets, which characterized lncRNAs in developmental stages as well as adult tissues in zebrafish. The similarities and differences within the three RNA-seq datasets with respect to the sample used, analysis protocols and lncRNAs identified have been discussed in a recent review on the field [19]. A merged annotation of these lncRNAs was made and this served as the template for the analysis of their expression levels in various datasets.

### Analysis of publicly available RNA-Seq data

**Fig 2:**
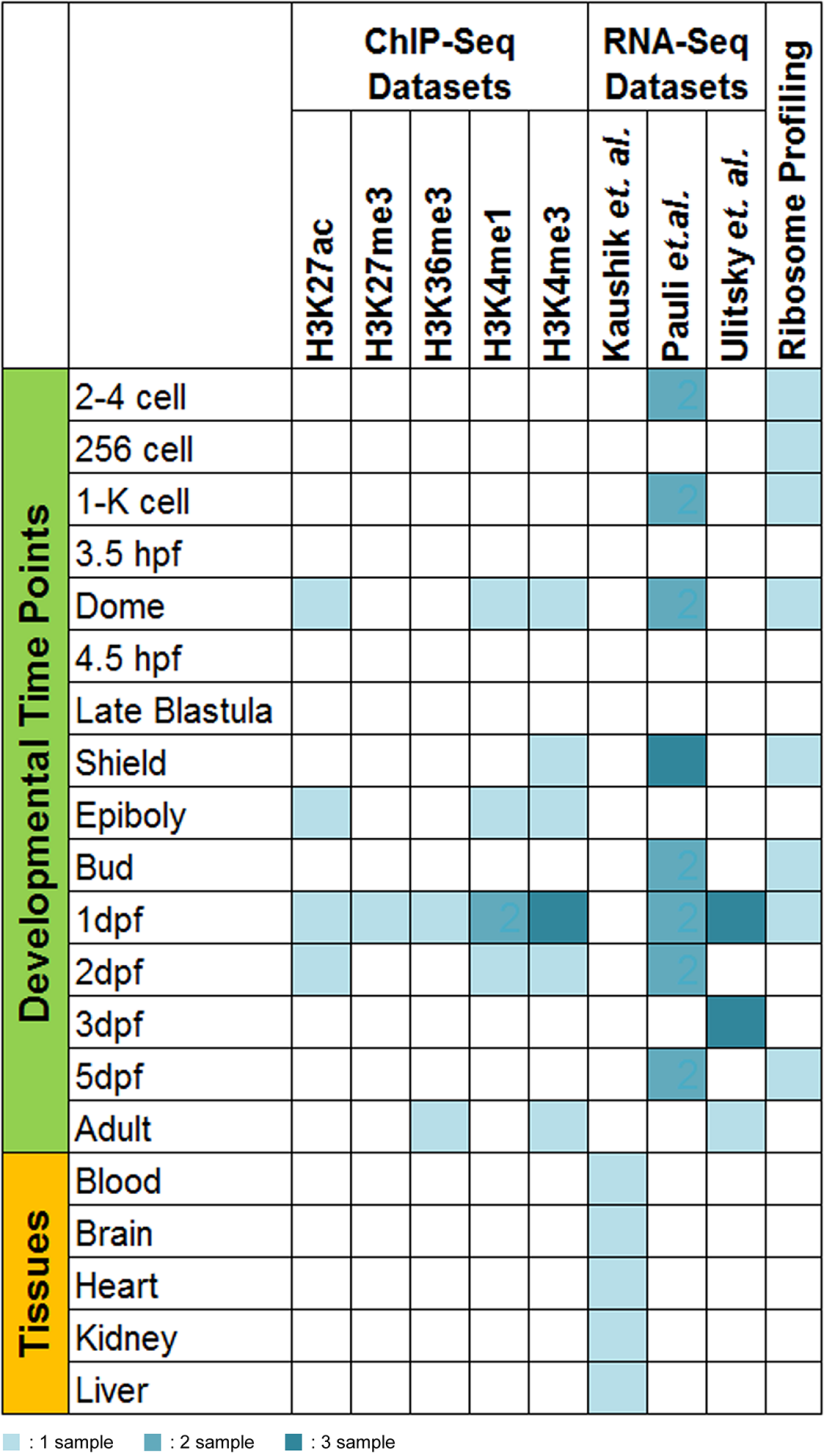
Matrix reporting sample count of various datasets used across different developmental time-points.

Raw RNA-Seq data for each study was downloaded from Sequence Read Archive (SRA) and the samples were analysed using the standard pipeline as detailed. The list of datasets used and descriptions are available as Table A in S1 File, Fig 2. TopHat was used for the alignment of reads to the reference genome (Zv9 genome assembly), which performs ultra fast short read mapping using bowtie based on exon–exon splice junctions [20]. Transcript assembly for different runs of each sample was done with cufflinks and the different assemblies were then merged for each sample using *cuffmerge*. Further downstream analysis for differential expression (DE) was carried out using *cuffdiff*. The lncRNAs were further classified and named on the basis of transcript type and their corresponding expression pattern.

### Mapping ChIP-Seq data on the lncRNome

Genome-wide ChIP-Seq datasets from five studies were retrieved from SRA and aligned to the reference genome of zebrafish using—Mapping and Assembly with Quality (MAQ) [21]. A complete list of the datasets used and descriptions is available in Supplementary Data I. The peaks were called using—Model-based Analysis of ChIP-Seq (MACS) [22] as described previously [23]. Histone modification marks included developmental time-points dome, shield, epiboly, 24hpf, 48hpf and adult stages of the zebrafish [18, 24–27].

### Integrating Ribo-Seq information

Zebrafish ribosomal profiles, as predicted by Chew *et*. *al*. [28], were also integrated in the database. Sequencing reads for ribosome-protected fragments for different developmental time-points spanning 2–4 cell, 256 cell, 1k cell, dome, shield, bud, 28 hpf and 5 dpf stages were retrieved from SRA. The dataset was preprocessed by removing the adapter sequences and discarding the reads that mapped to rRNA using Bowtie2 [29]. The remaining reads were then aligned to the zebrafish transcriptome and Zv9 genome assembly with TopHat2 [30] as described by Chew *et*. *al*. Coverage of Ribo-Seq reads across the zebrafish developmental time course can be analysed over the genome browser to check if the transcript can have any possible coding potential. In addition, the Open Reading Frames (ORFs) were predicted for each of the transcripts using getORF, which is available as part of the EMBOSS suite[31].

### Genomic variations and mutation information

A number of insertional mutagenesis approaches have been employed in zebrafish towards understanding gene functions by closely following up phenotypes using molecular methods. Retroviral genomic insertions from publically available datasets have also been included in the resource for each of the lncRNA [13, 32]. Apart from this, presence of important variations reported in dbSNP within the exonic regions of the lncRNAs was also checked and catalogued [33].

### Database design and architecture

The resource has been built in MySQL and the web interfaces have been coded in Perl-CGI. For each putative lncRNA, information related to the corresponding stage specific expression, open reading frames, the retroviral insertion maps and variant data has been compiled in different annotation tables and linked to provide a user-friendly interface. To explore the lncRNAs across entire genome, taking into consideration various available annotation marks, a genome browser has been embedded within the interface. Alignment maps of histone modification marks, ribosome profiling, expression levels, transcription factors and variations have been loaded into the browser. Tracks for RefSeq genes, ENSEMBL genes and the genes nearest to lncRNA are also added to enable accurate annotation and functional analysis of the transcripts taking into consideration the reports from all the associated studies.

## Results

### Database features and navigation

Information on a specific lncRNA is organized as a simple and browse-able interface, which can be searched using either their gene names, aliases, genomic loci or by the nearest protein-coding gene. To detail the salient features of the database, we describe the specific annotation for a well characterized lncRNA in zebrafish- megamind. The screenshot of the resource (Fig. A in S1 File) shows the annotation of the lncRNA. The expression profile of the lncRNA across developmental stages and tissues support the earlier observation that the lncRNA is highly expressed in brain among adult tissues, while provides additional information that it is developmentally regulated. The genome browser provides an option where the user can visualize the transcript in the context of various other integrated experimental datasets, including ribosome profiling data and histone modifications across developmental time points. Analysis suggests histone modification—H3K4me3 is closely associated with the lncRNA gene body, while activator mark H3K27ac shows association with the lncRNA promoter. The resource also provides a ready reference to genomic variations in the lncRNA loci and ready links to relevant citations describing the lncRNA and the sources of relevant datasets integrated.

An overview of the nearest gene and distance between the TSS of the corresponding lncRNA is provided in the list displayed on querying the input. Selecting a transcript provides detailed information on each transcript organized in the sections as detailed below:

### Transcript information

The database provides basic annotation of the lncRNA transcripts along with the nomenclature, aliases and genomic coordinates. The database content is largely derived from the three major and recent publications which include 691 lncRNAs predicted in early embryogenesis, 1,133 in late developmental stages and 442 from adult tissues. Each of the lncRNAs was further categorized into sense intronic, overlapping, intergenic and promoter associated depending on their genomic context in relation to protein-coding genes. Analysis revealed a total of 86 intronic, 485 overlapping, 309 promoter associated and 1,386 linc-RNAs. All the three studies showed a preponderance of intergenic lncRNAs. This observation could arise because the transcript overlaps with Ref-Seq protein coding genes were filtered for the annotation of lncRNAs.

### Genome Browser

The entire genome can be explored with an unparalleled speed through the genome browser featuring localized annotations for each of the transcript [34]. It displays various feature tracks that include exonic regions, nearest gene, variations, expression levels in different stages and five epigenetic marks, transcription factors and ribosome profiles across different stages from various samples simultaneously in a single panel.

### Expression and regulation

The availability of a catalog of lncRNAs encompassing all the annotations and the expression levels of the transcripts from RNA-Seq data offers a unique opportunity towards creating a spatiotemporal map of gene expression in lncRNAs. In addition to the track displayed in genome browser, expression levels across ten developmental time points and five adult tissues are represented graphically with log_10_ FPKM values plotted across different stages. This section details the conditions in which the lncRNA is highly expressed.

A number of recent reports have characterized the promoter epigenetic marks of lncRNAs and have suggested that the promoter epigenetic marks in lncRNAs are similar to that of protein coding genes [35]. Drawing parallels, it would be imperative to understand the epigenetic marks associated with lncRNAs. Histone modification marks encompassing H3K27ac, H3K36me3, H3K4me1, H3K4me3 and H3K27me3 reported in zebrafish across different developmental time points have been integrated and provided in the genome browser. For ease of interpretation, activator marks are shown in green color while the repressor marks are depicted in red color. In addition, ChIP-Seq datasets encompassing a number of critical transcription factors have also been integrated in the genome browser. This includes transcription factors such as Nanog-like, Mxtx-2, gata-1, Sox-2, Pou5f1, Cdx-4 and Sal-4 [36–39].

### Mutant information

The resource provides an easy access to information regarding mutants thereby aiding researchers to study them in detail towards understanding the biological mechanisms and phenotypes associated with the particular lncRNA. A systematic mapping of a total of 15,223 publicly available retroviral insertions from ZFIN [13] showed a total of 111 insertions mapped to 126 lncRNA transcripts. A set of 156 insertions reported in ZETRAP have also been included in the resource as a track in the browser [32].

Apart from this, in context to the queried transcript the predicted open reading frames and important variations falling within the exons and references pointing to relevant literature information are also provided. In addition, the experimental datasets for ribosome profiling during developmental time-points are also provided as a brows-able track on the genome browser.

In summary, the resource thereby allows the study of zebrafish as a model organism with a broad perspective taking into consideration the genomic, transcriptomic and epigenetic context. A comparative analysis of the features of zflncRNApedia vis-à-vis other two major resources–ZFIN and lncRNAdb, is reported in Fig. B in S1 File suggesting unique salient features in the resource. The list of predicted transcripts, corresponding expression levels, variations and retroviral insertion maps have been provided for download at the home page as tab-delimited text files. In addition, the compendium of lncRNA annotations could be visualized on UCSC genome browser as a track hub.

## Conclusion and Discussion

Long non-coding RNAs are increasingly shown to play intricate roles in critical biological functions, though a large majority of members of this class are poorly characterized and functionally annotated. The annotation of lncRNA repertoire in zebrafish largely comes from recent deep transcriptome sequencing approaches from three complementary studies. Each of these studies identified a distinct lncRNome encompassing distinct developmental time-points and adult tissues. It was thus imperative to have an integrated resource, putting together evidence from multiple experiments as a starting point to understand and prioritize lncRNAs for biological studies. In addition, the spatiotemporal map of gene expression of these lncRNAs would provide clues towards their potential functional characteristics and regulatory dependence. To this end, we have compiled all relevant datasets on zebrafish lncRNAs to provide a user-friendly online resource—zflncRNApedia. Unlike any other available resource, zflncRNApedia enables easy analysis of spatio-temporal expression patterns of lncRNAs in context to various regulatory marks that include histone modifications and transcription factors.

With the reducing cost, nucleotide sequencing is becoming a common approach to study transcriptome dynamics. We anticipate discovery of newer lncRNAs from deep sequencing studies and subsequent mapping of insertion and ENU mutants to these transcripts. zflncRNApedia would be regularly updated with the flow of new information to explain a number of phenotypes and to enable molecular characterization of functions with the enriched data. As evident from the diversity of nomenclature followed by individual studies, a centralized database would enable a systematic and standard process of gene annotation for lncRNAs. We also foresee significant enrichment in the molecular, functional and phenotypic information on long non-coding RNAs as many of them get molecularly and functionally probed.

## Supporting Information

S1 File Table A. Description and source of datasets used in compiling the resource. Fig. A. Screenshot of zflncRNApedia featuring the different sections of the database for a candidate lncRNA–*Megamind*. Fig. B. Comparative analysis of zflncRNApedia with ZFIN and lncRNAdb based on database features and content.
(RAR)
